# Frameworks for Implementation, Uptake, and Use of Cardiometabolic Disease–Related Digital Health Interventions in Ethnic Minority Populations: Scoping Review

**DOI:** 10.2196/37360

**Published:** 2022-08-11

**Authors:** Mel Ramasawmy, Lydia Poole, Zareen Thorlu-Bangura, Aneesha Chauhan, Mayur Murali, Parbir Jagpal, Mehar Bijral, Jai Prashar, Abigail G-Medhin, Elizabeth Murray, Fiona Stevenson, Ann Blandford, Henry W W Potts, Kamlesh Khunti, Wasim Hanif, Paramjit Gill, Madiha Sajid, Kiran Patel, Harpreet Sood, Neeraj Bhala, Shivali Modha, Manoj Mistry, Vinod Patel, Sarah N Ali, Aftab Ala, Amitava Banerjee

**Affiliations:** 1 Institute of Health Informatics University College London London United Kingdom; 2 Charing Cross Hospital Imperial College Healthcare NHS Trust London United Kingdom; 3 Division of Anaesthetics, Pain Medicine, and Intensive Care, Department of Surgery and Cancer Faculty of Medicine Imperial College London London United Kingdom; 4 School of Pharmacy University of Birmingham Birmingham United Kingdom; 5 University College London Medical School University College London London United Kingdom; 6 Department of Population Health Sciences King’s College London London United Kingdom; 7 eHealth Unit Research Department of Primary Care and Population Health University College London Medical School London United Kingdom; 8 University College London Interaction Centre University College London London United Kingdom; 9 Diabetes Research Centre Leicester General Hospital University of Leicester Leicester United Kingdom; 10 Department of Diabetes and Institute of Translational Medicine University Hospital Birmingham Birmingham United Kingdom; 11 Division of Health Sciences Warwick Medical School University of Warwick Coventry United Kingdom; 12 Patient and Public Involvement Representative DISC Study (UK) United Kingdom; 13 University Hospitals Coventry and Warwickshire Coventry United Kingdom; 14 Health Education England London United Kingdom; 15 Hurley Group Practice London United Kingdom; 16 Queen Elizabeth Hospital Birmingham University Hospitals Birmingham NHS Foundation Trust Birmingham United Kingdom; 17 Institute of Applied Health Research University of Birmingham Birmingham United Kingdom; 18 Warwick Medical School University of Warwick Coventry United Kingdom; 19 Department of Diabetes and Endocrinology Royal Free London NHS Foundation Trust London United Kingdom; 20 Department of Access and Medicine Royal Surrey NHS Foundation Trust Guildford United Kingdom; 21 Department of Clinical and Experimental Medicine Faculty of Health and Medical Sciences University of Surrey Guildford United Kingdom; 22 Institute of Liver Studies King’s College Hospital NHS Foundation Trust London United Kingdom

**Keywords:** eHealth, framework, cardiometabolic, health inequalities, health inequality, health technology, ethnicity, minority, digital health, review, cultural, diverse, diversity, cardiology, metabolism, metabolic

## Abstract

**Background:**

Digital health interventions have become increasingly common across health care, both before and during the COVID-19 pandemic. Health inequalities, particularly with respect to ethnicity, may not be considered in frameworks that address the implementation of digital health interventions. We considered frameworks to include any models, theories, or taxonomies that describe or predict implementation, uptake, and use of digital health interventions.

**Objective:**

We aimed to assess how health inequalities are addressed in frameworks relevant to the implementation, uptake, and use of digital health interventions; health and ethnic inequalities; and interventions for cardiometabolic disease.

**Methods:**

SCOPUS, PubMed, EMBASE, Google Scholar, and gray literature were searched to identify papers on frameworks relevant to the implementation, uptake, and use of digital health interventions; ethnically or culturally diverse populations and health inequalities; and interventions for cardiometabolic disease. We assessed the extent to which frameworks address health inequalities, specifically ethnic inequalities; explored how they were addressed; and developed recommendations for good practice.

**Results:**

Of 58 relevant papers, 22 (38%) included frameworks that referred to health inequalities. Inequalities were conceptualized as society-level, system-level, intervention-level, and individual. Only 5 frameworks considered all levels. Three frameworks considered how digital health interventions might interact with or exacerbate existing health inequalities, and 3 considered the process of health technology implementation, uptake, and use and suggested opportunities to improve equity in digital health. When ethnicity was considered, it was often within the broader concepts of social determinants of health. Only 3 frameworks explicitly addressed ethnicity: one focused on culturally tailoring digital health interventions, and 2 were applied to management of cardiometabolic disease.

**Conclusions:**

Existing frameworks evaluate implementation, uptake, and use of digital health interventions, but to consider factors related to ethnicity, it is necessary to look across frameworks. We have developed a visual guide of the key constructs across the 4 potential levels of action for digital health inequalities, which can be used to support future research and inform digital health policies.

## Introduction

Individuals of an ethnic minority background constitute at least 14% of the UK population [1] and have an increased risk of type 2 diabetes [2] and cardiovascular disease [3] (together, also known as cardiometabolic disease), particularly South Asian and Black individuals. Even before, but particularly during, the COVID-19 pandemic, digital health interventions became important in the education, prevention, diagnosis, treatment, and rehabilitation [4,5] of diseases such as cardiometabolic disease [6,7].

Whether via smartphones, websites, or text messaging, digital health interventions need to be culturally competent (ie, able to meet the needs of users with diverse values, beliefs, and behaviors) to be accessible to all [8,9], but the effectiveness of digital health interventions may vary across different groups (by age, clinical need, socioeconomic, or other factors) [7]. Moreover, unequal access to hardware, software, and the internet, as well as variations in digital literacy, create a digital divide through which digital health interventions could exacerbate existing socioeconomic, educational, and health inequalities [10,11]. Therefore, digital health interventions, similar to other health interventions, require robust evaluation before and after implementation, by using frameworks that take into account society-level (eg, political context, interorganizational networks), system- or organization-level (eg, organizational capacity and engagement), and individual (eg, literacy, financial resources) factors. Existing frameworks include those adapted from other fields [12,13], as well as those developed specifically for health and health care technology [14]. Despite multiple ways of analyzing health inequalities [15], frameworks have often overlooked the experiences of ethnic minority populations. Given the excess cardiometabolic burden faced by ethnic minority groups, digital health interventions designed for cardiometabolic disease are an important area of study.

This scoping review aims to identify existing frameworks, models, or theories that address (1) implementation, uptake, and use of digital health interventions by end users; (2) health interventions in ethnically or culturally diverse populations; or (3) interventions for cardiometabolic disease. For identified frameworks, we examine the extent to which they include and how they address health inequalities, specifically regarding ethnicity and relevance to ethnic inequalities in cardiometabolic disease.

## Methods

### Search Strategy and Selection Criteria

We conducted this review in accordance with PRISMA-ScR (Preferred Reporting Items for Systematic Reviews and Meta-Analyses for Scoping Reviews) guidelines (Multimedia Appendix 1). We included papers that presented a new, revised, or adapted framework that could be used to understand either factors in: the adoption and acceptance of digital health; or cardiometabolic interventions; or sociodemographic inequalities in health (Multimedia Appendix 2). We considered frameworks to be any models, theories, or taxonomies. There are multiple definitions of implementation and the technology acceptance lifecycle [16,17]. We focused on 3 stages: implementation (putting interventions to use within a setting) [17], uptake (adoption by end users), and use (sustained use and acceptance) [16]. We excluded frameworks aimed at delivery processes, technology development processes, or economic assessments. Given the extensive literature on frameworks for technology adoption, only papers that presented frameworks that have been designed or adapted to health and care settings were included. There was no limit on publication date.

### Information Sources

SCOPUS, PubMed, EMBASE, and Google Scholar were searched electronically in April 2021 (by MR). Gray literature was identified via OpenGrey [18] and the New York Academy of Medicine Grey Literature Report [19].

### Search

An initial keyword search (“digital” AND “health” AND “ethnicity” AND “cardiometabolic” AND “framework”) demonstrated that there was no existing systematic or scoping review that addressed ethnic digital health inequalities. The 3 areas of interest for review were used to define relevant keywords for the search strategy (Multimedia Appendix 3).

### Study Selection

Search result records were imported into Rayyan (Qatar Computing Research Institute) after removing duplicate records. Title and abstract screening against inclusion and exclusion criteria were conducted by a team (AC, AGM, JP, LP, MB, MM, MR, PJ, ZTB), with 2 rounds of testing in which any queries were discussed. The guide for interpretation of the inclusion criteria that was developed via this iterative approach can be found in Multimedia Appendix 4. Additional frameworks identified at the abstract screening stage were searched for and added to the full-text review (Multimedia Appendix 5). Full texts were reviewed (by MR) if abstracts lacked sufficient information. The final selection was made by 2 authors (MR and LP); disagreements in study selection were resolved by discussion until consensus was reached, or with a third reviewer (ZTB) when it was not reached.

### Data Analysis

Data charting was piloted on 10 randomly selected papers and refined to ensure consistency across researchers (categories of information are set out in Multimedia Appendix 6). Data charting was repiloted on 10 additional studies and after a final review to ensure agreement in information extracted and summarized, the remainder of the papers were charted. Citation details, evidence type, framework context, framework focus, and framework beneficiary were charted. Qualitative analysis was conducted. Data are reported according to PRISMA-ScR [20]. Papers were assessed for the degree to which they considered factors related to inequalities: this was defined broadly to include racial, ethnic, or cultural diversity; health inequalities; digital inequalities; or social determinants of health.

## Results

### Scoping Review

**Figure 1:**
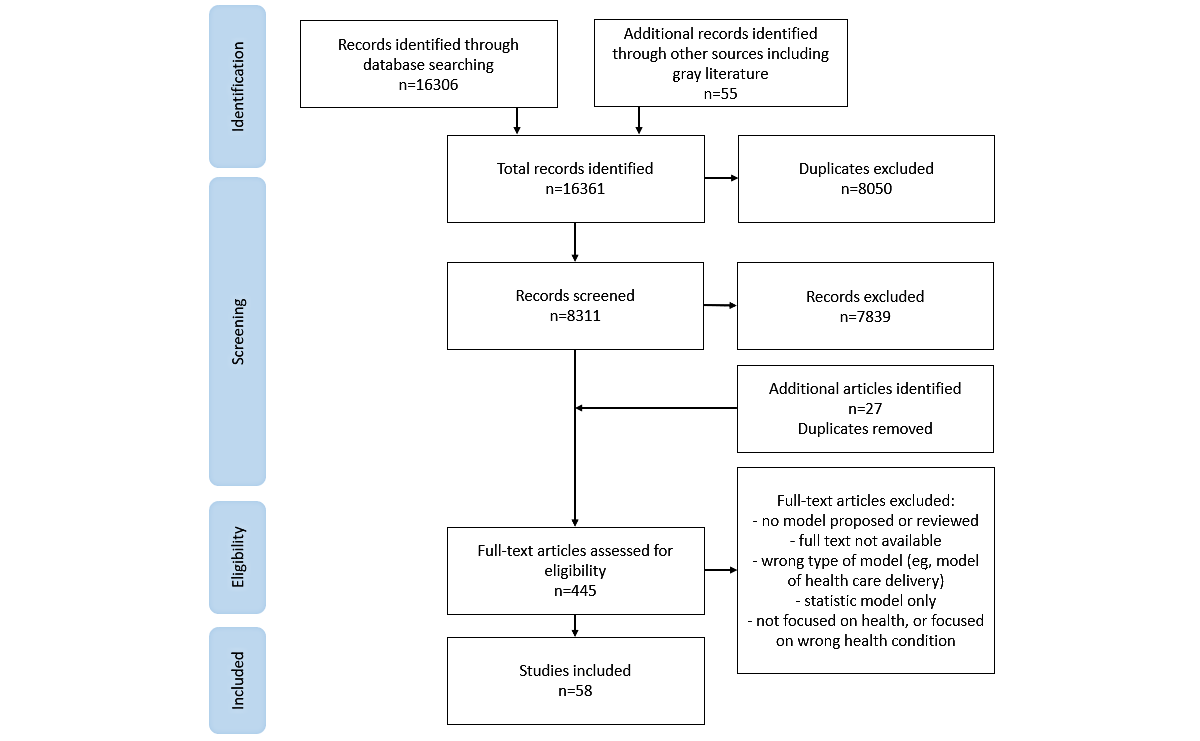
Paper selection flowchart.

A total of 7830 unique records were identified. A total of 58 papers were included (Figure 1; Multimedia Appendix 7), of which 32 papers included adapted or extended existing frameworks. A majority included the Technology Acceptance Model [21-37] or the Unified Theory of Acceptance and Use of Technology [26,27,38-43]. New frameworks, developed from the review and synthesis of existing frameworks or from empirical research, were proposed by 26 papers [14,15,44-67]. First author institution was listed in Europe, North America, or Australia for the majority of papers (n=39) [14,23,24,31-33,35,37,39,43,44,46-48,51-55,58-77]; Asia or the Middle East (n=13); and South Africa (n=2) [50,57]. The remaining had first authors with affiliations in more than one country [15,26,27,36,56]. Many papers did not specify the geographic location in which the framework was designed for use or testing [14,15,24,27,31,35,44-46,49-55,58-61,68,69, 71,74,75] (n=25); of those that did, the majority (n=14) were developed or tested in Europe, North America, or Australia [37,39,43,47,62-67,70,72,76,77].

The majority of frameworks had digital health interventions or health technology (such as electronic health records, or remote monitoring) as the only or key focus (n=39). Fifteen of the remaining frameworks considered at least two of digital health interventions, health inequalities and ethnicity, or cardiometabolic disease. The purpose of most frameworks was to understand factors related to the adoption, acceptance, and use of digital health technology (n=43), with the remaining frameworks (n=15) considering health inequalities, chronic disease management, and evaluation of interventions. In the majority of papers, the end user who was likely to benefit from the application of the framework was either a patient or member of the public (eg, as targets for interventions for disease prevention or management) (n=33) or a clinician (n=5). Seven frameworks focused on the intervention or technology itself. The remaining frameworks had no specific end user or covered a combination of benefits.

### Extent of Inclusion of Health Inequalities in Existing Frameworks

Over half of the papers that showed no or limited inclusion of inequalities (26/36) did not address inequalities in either the body text or the framework themselves. A few papers (n=7) acknowledged the wider socioeconomic context in the paper or included a high-level reference to social or contextual factors that might influence uptake and use of health technology, for example, by including the factor *broad context* [44]. Another group of frameworks took digital access into account within the *facilitating conditions* construct, based on either the Technology Acceptance Model [28] or the Unified Theory of Acceptance and Use of Technology [41,43]. Many were focused on the factors affecting adoption and use in specific populations, such as older adults (n=6), the workforce (n=8), or in Asian or low- and middle-income contexts (n=5) (Table 1).

A few frameworks took the specific challenges of mobile health (mHealth) readiness [56], adoption [26,57], acceptance [23], and impact on access to care [32] in low- or middle-income countries into account; these frameworks were assessed as having limited applicability to the specific challenges of multiethnic populations in Western countries. Some frameworks that focused on understanding patient or public acceptance of and engagement with digital health interventions considered how these may be affected by factors related to health or digital inequalities, for example, tech generation (experience of individuals of different age groups of different technologies), health literacy, and education [58]; demographic, psychological, physical, and social factors [59]; or personal lifestyle factors [60] (Table 2). Many papers that looked specifically at ethnic inequalities in health frameworks included ethnicity in the *demographic factors* element of the framework itself [15,25,59,61,62,74-76] or discussed ethnicity in the accompanying text [63-65]. Notably, Schillinger [65] discussed the limitations of current research on health literacy and known racial and ethnic health disparities [65]. Only 3 frameworks (Table 2) focused on the mechanisms through which ethnicity impacts health and engagement with interventions [25,66,76].

**Table 1:** Frameworks with no or limited consideration of ethnic and social inequalities in health.

Reason for which papers were deemed to have no or limited consideration and the key focus of the framework				Papers (n=36)		
				Reference		n
**Does not address health or digital inequalities (population)**						
	Older adults or elderly populations			[21,31,36,45,68]		5
	Health care professionals			[27,40,46-48,69]		6
	Workplace or workforce			[34,42]		2
	South Asian and low- and middle-income contexts			[21,29,30,33]		4
	Other			[24,39,49-52,70,71]		8
	Review paper			[35]		1
**Acknowledgment of contextual factors in the paper only**						
	Digital cardiovascular prevention			[37]		1
	Implementation effectiveness			[53]		1
**High-level factoring of the wider context in the framework figure**						
	Engagement with health apps			[72]		1
	Integration of health interventions into health systems			[44]		1
**High-level factoring of social factors or access into the framework**						
	**Digital access considered within the facilitating conditions construct of the Technology Acceptance Model or the Unified Theory of Acceptance and Use of Technology variant**					3
		Electronic health record adoption	[43]			
		Older adults	[41]			
		Tested in Pakistan	[28]			
	**Model includes broadly defined factors such as sociodemographic factors**					3
		National culture differences in acceptance	[73]			
		Telehealth in chronic disease intervention design and evaluation	[54]			
		Implementation planning and evaluation	[55]			

**Table 2:** Frameworks that show some or detailed consideration of ethnic and social inequalities in health.

Reason for which papers were deemed to show some or detailed consideration and the key focus of the framework				Papers (n=22)		
			Reference		n	
**Model aimed at global health inequalities or developed in low- or middle-income countries**						
	mHealth^a^ adoption in developing world		[26,57]		2	
	mHealth readiness, developed in rural Bangladesh		[56]		1	
	mHealth contributions to care access, sub-Saharan Africa		[32]		1	
	mHealth interventions targeted at low-literacy end users in resource-limited settings		[23]		1	
**Includes factors related to health or digital inequalities**						
	Acceptance of remote patient management		[58]		1	
	Engagement and recruitment to digital health intervention		[59,60]		2	
	Nonadoption, Abandonment, Scale-up, Spread, and Sustainability framework		[14]		1	
**Framework aims to address health inequalities or to be used in populations facing health inequalities**						
	**Health inequalities**				3	
		A Conceptual Framework for Action on the Social Determinants of Health	[15]			
		Community Chronic Care Model	[77]			
		Conceptual Framework for the Pathways that Connect Social Determinants of Health, Health Literacy and Health Disparities	[65]			
	**Digital health and access or inequalities**				6	
		eHealth Equity Framework	[74]			
		Digital Health Equity Framework	[75]			
		The Updated Integrative Model of eHealth Use	[63]			
		Modeling the process of using an eHealth tool by people vulnerable to social health inequalities	[61]			
		Culture-centered Technology Acceptance Model	[25]			
		Pathways of access, use, and benefit from digital health services	[64]			
	**Cardiometabolic disease and inequalities**				4	
		Conceptual framework for understanding the development and role of financial barriers for patients with cardiovascular-related chronic diseases	[67]			
		A Gender-Centered Diabetes Management Education Ecological Framework	[76]			
		Diabetes in Ageing and Diverse Populations	[66]			
		Workforce Evidence-Based model for diabetes	[62]			

^a^mHealth: mobile health.

### How Frameworks Address Health Inequalities

**Figure 2:**
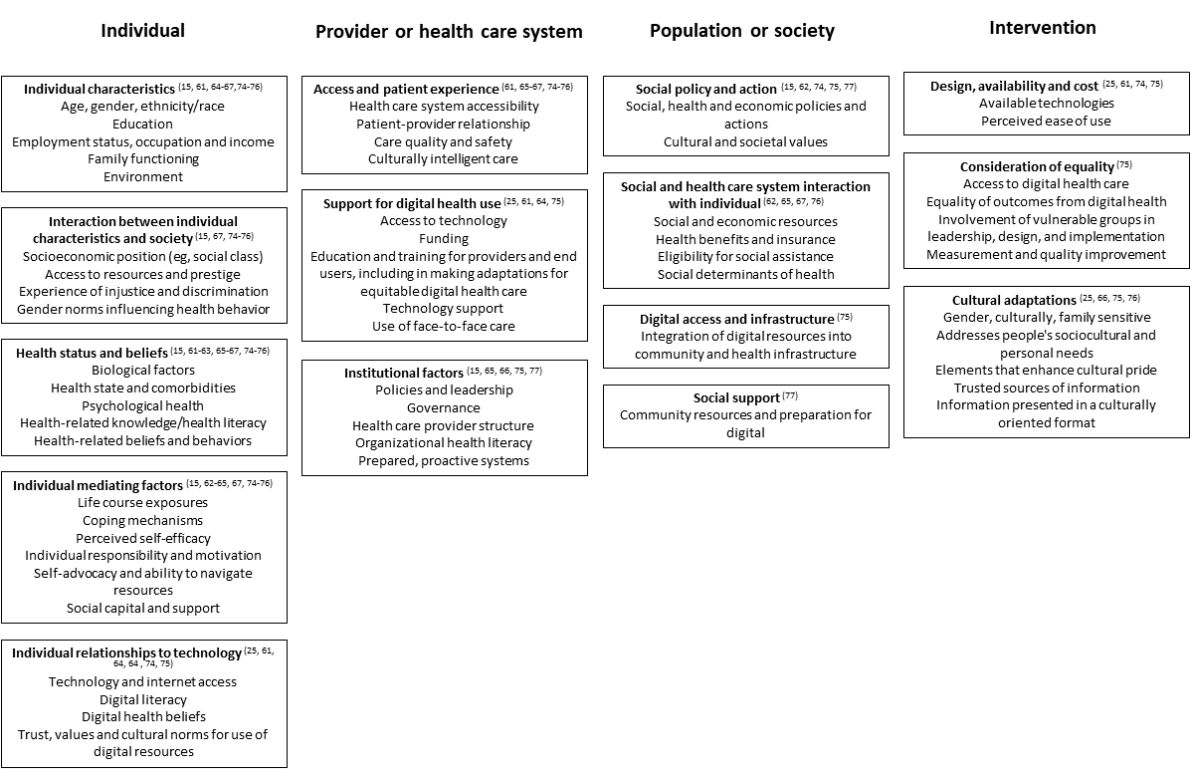
Guide showing how framework constructs that consider inequalities map onto the 4 levels of action.

We identified 13 frameworks that explicitly aimed to understand or address general health inequalities [15,65,77], health inequalities in relation to the management of cardiometabolic disease [62,66,67,76], digital health equity [61,63,64,74,75], or recommendations on how to culturally tailor digital health approaches [25] (Table 3). Key factors or constructs in these frameworks [15,25,61-67,74-77] could be mapped to the 4 levels of action in which digital health care is seen to operate—society or population, health care system, intervention, and individual (Figure 2)—and 5 frameworks included factors in all 4 levels, for example, individual health status and beliefs, support for digital health use, social policy and action, and cultural adaptations of the intervention [25,66,74-76]. The wide scope of factors included in these frameworks reflects the diversity of theoretical approaches used, for example, adaptation of an existing model of social determinants of health to digital health [74,75], adaptation of existing models such as the Technology Acceptance Model for interventions or innovation [25,63,77], and the development of novel frameworks through methods such as grounded theory or thematic analysis [61,62,66,67] (Table 3).

Some frameworks delineated the interaction between these levels to account for how health inequalities occur [15,65,77]. Such frameworks tended to focus on the top-down processes by which societal and system factors filter down to affect health outcomes [15,65,77]. For example, the Community Chronic Care Model [77] was used to demonstrate how community resources and health care provider systems contribute to improved community-wide health outcomes. Schillinger [65] brought together research from multiple disciplines, such as epidemiology, anthropology, and public health, to describe two routes through which social determinants of health act on health outcomes and health disparities: unequal distribution of resources and the health care systems themselves.

We identified 3 frameworks [63,74,75] that were developed as tools to understand and address the potential role of digital health interventions in exacerbating existing health inequalities. The eHealth Equity Framework [74], based on the World Health Organization’s Commission on Social Determinants of Health conceptual framework [15], incorporates technology into the macro socio-techno-economic-political context with intermediary determinants of health care access and use, such as material circumstances, social capital, and literacy. Similarly, the Digital Health Equity Framework [75] integrated digital determinants of health and digital health equity into known health equity factors based on previous work [78]. The Updated Integrated Model of eHealth Use describes how social determinants of health impact user interactions with health technologies and health outcomes [63].

Three frameworks targeted the design and implementation of digital health interventions. In 2 papers [61,64], the use of digital health tools by people vulnerable to social inequalities and opportunities to identify and address barriers were discussed. In another paper [25], the extension of the Technology Acceptance Model, by integrating Community Infrastructure Theory, was described and approaches to engage with marginalized populations were tested.

We found 4 frameworks relevant to cardiometabolic disease. Two frameworks looked at socioeconomic factors affecting health inequalities: one focused on supporting health care professionals to identify and support at-risk groups [62], and the other considered the role of financial barriers on outcomes for patients with cardiovascular-related chronic diseases [67]. Two frameworks aimed to improve outcomes for diabetes in specific ethnic minority groups: older South Asian adults in the United Kingdom [66] and Black men in the United States [76].

**Table 3:** Frameworks that consider equity in digital health or cardiometabolic disease intervention.

Framework or key focus			Reference		Purpose		Theoretical basis		Intended audience
**Digital health equity (conceptual)**									
	eHealth Equity Framework	[74]		Apply a health equity approach within eHealth		World Health Organization Conceptual Framework for Action on the Social Determinants of Health [15]		Public health, research, policy, health technology development	
	Digital Health Equity Framework	[75]		Identify the digital determinants of health and their links to digital health equity		Health equity measurement framework [78]		Research, health (service) implementation	
	Updated Integrative Model of eHealth Use	[63]		Understand how (digital and health) literacy contributes to health and well-being		Integrative Model of eHealth Use [79]		Health communication, public health	
**Equitable digital health services**									
	Pathways of access, use, and benefit from digital health services	[64]		Map key factors influencing digital health service outcomes		Frameworks of access to health services		Research, policy, health services, and public health	
**Equitable digital health intervention design**									
	Modeling the process of using an eHealth tool by people vulnerable to social health inequalities	[61]		Identify stages of the process of using an eHealth tool that can account for reducing barriers for those at risk of social health inequalities		Structural Influence Model		Research, health technology development	
	Culture-centered Technology Acceptance Model	[25]		Describe factors that account for people's social and cultural needs when considering technology acceptance		Technology Acceptance Model [80]		Policy, health technology, or intervention development	
**Reducing impact of inequalities in patients with cardiometabolic disease**									
	Conceptual framework for understanding the development and role of financial barriers for patients with cardiovascular-related chronic diseases	[67]		Understand the patient experience of financial barriers and impact on behavior and clinical outcomes (in relation to chronic disease)		None specified		Research, clinical, policy	
	Workforce Evidence-Based model for diabetes	[62]		Recognize and manage the complex needs of individual patients with chronic disease		None specified		Clinical, research, health education, health service, and workforce planning	
	Diabetes in Ageing and Diverse Populations	[66]		Map how links between cultural competency, comorbidity and stratification, and access can contribute to effective diabetes care for aging and diverse populations		Realist review approach, underpinned by the theme of individualized care		Research	
	A Gender-Centered Diabetes Management Education Ecological Framework	[76]		Incorporate gender into an understanding of variables that affect diabetes health outcomes		Key focus is theories of gender		Research (diabetes education)	
	Community Chronic Care Model	[77]		Map how community and health care provider systems interact with other influences to improve community-wide health outcomes and eliminate health disparities		Chronic Care Model, concepts of community		Community and health care provider organizations, research, clinical	

### Ethnic Inequalities in Cardiometabolic Disease

Nine papers recommended solutions to increase the adoption and acceptance of interventions in ethnically or culturally diverse populations, with some focusing on cardiometabolic disease. The Workforce Evidence-Based model for diabetes [62] was developed to meet the need for tailored management for a diverse patient population, by guiding health professionals in determining which patients may require additional support. In the culture-centered Technology Acceptance Model [25], a range of individual and intervention attributes that can impact acceptance, such as enhancing cultural pride or using presenters from the community to increase trust, are identified.

The Community Chronic Care Conceptual Model was used to show how community resources and health care provider systems can interact with other factors to impact community-wide health outcomes, with examples of direct action, such as increasing community health professional training targeted at reducing amputations in African-American men with diabetes [77]. Other recommendations for action included video-based information for the public [63,77], internet training, and meaningful involvement in patient groups from co-design to implementation [63,75]. However, working with South Asian people with diabetes in the United Kingdom, Wilkinson et al [66] noted the need for further data to understand the effectiveness of cultural adaptations and approaches to culturally competent care, such as peer support. Crawford and Serhal [75] also reiterated the need for additional data collection around health inequalities to implement and evaluate digital health through an equitable lens.

## Discussion

### Principal Findings

We identified 58 frameworks relevant to digital health adoption that address health inequalities and cardiometabolic interventions. Several frameworks were found to consider health inequalities in digital health interventions and inequalities in cardiometabolic disease, but none covered all 3 areas of interest. Less than half (n=22) addressed health inequalities in detail; the remainder did not address health or digital inequalities at all or included only a high-level factor in the body text of the paper or as a framework construct (such as “differentiated by national culture” [73] or “wider social and health system” [54]). We identified 3 models for understanding the digital determinants of health equity [74,75] and 3 frameworks that describe factors related to implementation, uptake, and use of health technologies [25,61,64].

Where health inequalities were considered, they were broadly related to social theory, and more specifically, the social determinants of health, which is described as “the causes of the causes” [81] of health inequality. For example, in the papers [15,75] describing the Digital Health Equity Framework and the Commission on Social Determinants of Health Conceptual Framework, it is highlighted that the health system itself acts as a social determinant of health. In the paper [74] that presented the eHealth Equity Framework, it is argued that technology should be integrated into models of health, in much the same way that the role of social structures is integrated in models of health and well-being outcomes.

In the majority of frameworks, ethnicity was considered under this broad banner of social determinants of health, rather than as a separate construct [15,25,59,61,62,74-76]. While this approach is a useful starting point when considering the factors related to implementation, uptake, and use, a more detailed approach is necessary when considering complex social, educational, and cultural factors relevant in ethnic minority groups for the design, implementation, and evaluation of digital health interventions. For example, a recent report highlighted the specific experiences of people from an ethnic minority background using the National Health Service (NHS) in England, including lack of trust, fear of discrimination, experiences of culturally insensitive behavior, communication barriers, and racism [82]. There is also evidence of worse outcomes for ethnic minority populations with specific digital health approaches, for example, differences in referrals to urgency and emergency care services by the NHS Direct telephone service [82]. We found only 3 frameworks that explicitly considered these factors [25,66,76]. In producing the culture-centered Technology Acceptance Model, Guttman and colleagues [25] describe the experiences of Ethiopian immigrants in the health care system in Israel and set out an iterative design process for a health website that took into account views from community groups and individuals. Culture-centered constructs, such as “elements that enhance cultural pride” and “addresses people’s sociocultural and personal needs” emerged from this research [25]. These constructs represent motivations to use the website beyond health information, for example, pride in traditional, cultural, and language identity, and benefits such as improving intergenerational communication [25]. Culturally tailored designs have been found to be important in digital health interventions for ethnic minority and other underserved populations [83].

Two frameworks were specifically designed in the context of ethnic differences in diabetes care and outcomes. Knowledge gained from these can be applied to other chronic health conditions and to the design and implementation of digital health services. Wilkinson and colleagues [66] did not identify any studies that focused on older people from a South Asian background in a review of literature on diabetes care. Their theoretical framework draws relationships between key concepts emerging from the literature: cultural stratification and comorbidities, cultural competency, and access [66]. The Gender-Centered Diabetes Management Education Ecological Framework takes a more detailed approach to address disparities in diabetes outcomes for Black men in the United States by placing diabetes management education into a broad context that includes demographic characteristics, gender roles, and family situation. While developed in one particular group, these constructs are applicable to understanding health management in other ethnic minority groups; for example, specific barriers to exercise have been identified in South Asian women with diabetes and cardiovascular disease, including family obligations, fears about women going out alone, lack of single-sex exercise facilities [84], and perceptions of taking time to exercise as being “selfish” and taking women away from their “daily work [85].”

### Comparison With Prior Work

It is necessary to consider health disparities in research on health technology, particularly in understanding the role of technology in exacerbating or addressing inequalities, and in the design and evaluation of interventions [86]. Approaches including defining common terms and proposing standardized language and measurement tools [16], mapping concepts of engagement with digital behavior change interventions [59], and describing commonly used frameworks in clinicians’ adoption of mHealth [27] have been used to review frameworks for the uptake and use of digital health interventions. Recently, reviews on equitable approaches to research [87] and use [88] of health portals have examined digital health equity at the intervention level. Researchers have also responded to the need for equitable approaches to virtual care provision (eg, access to phone or video consultations) highlighted during the COVID-19 pandemic [89,90], including adaptation of the Nonadoption, Abandonment, Scale-up, Spread, and Sustainability framework [14] to include digital inclusion as a concept that contributes to the *patient* domain [90].

As digital health approaches become embedded in national health strategies, there is also a need for the application of frameworks to ensure equitable digital health implementation in ethnically and culturally diverse populations. The NHS is promoting digital services and tools in England [91], including for cardiometabolic disease, such as a digital pilot of the NHS Diabetes Prevention Programme [92] and a cardiology digital playbook that promotes digital tools to support patients remotely [93]. Furthermore, the adoption of digital health interventions was actively encouraged to mitigate the risk of face-to-face interaction during the pandemic [94], and going forward, digital health interventions are seen as adoption of innovation to provide cost-effective outcomes in health [95]. However, digital exclusion has the potential to exacerbate health inequalities, both directly (reduced access to services and resources) and indirectly (access to wider determinants of health, such as housing or occupation opportunities) [96]. The frameworks identified in this scoping review and the guide to the key constructs they contain (Figure 2) can be used as tools to identify the individual, technological, and contextual factors that influence the direct routes between digital and health inequalities.

### Strengths and Limitations

We aimed to explore the breadth of potential frameworks that were applicable to understanding inequalities in digital health uptake and use. The configurative approach to a scoping review generates or explores theories, rather than aggregating data to test theories [97]. Taking an iterative approach also allows inclusion and exclusion criteria to be refined through the course of the review [98]. In this case, with an unknown literature base regarding digital health inequalities, we were able to further refine inclusion criteria during the full-text review to exclude a number of papers that focused on statistically testing minor variations of the Technology Acceptance Model. However, scoping reviews do not usually undertake formal quality appraisal [98]; therefore, synthesizing the results was difficult because of the range of frameworks identified. In a review of Technology Acceptance Model adaptations alone, a high degree of study heterogeneity was identified [12]. Additionally, there was a lack of standardization of terms, with the terms *acceptance*, *adoption*, and *acceptability* being used interchangeably. We took an inclusive approach when considering the use of such terminology [12,16].

### Future Directions

Beyond the scope of the review, other papers were identified during the screening process, which could have some relevance for the process of design and implementation of digital health interventions, for example, the RESET (relevance, evidence base, stages of intervention, ethnicity and trends) tool to adapt health promotions to meet the needs of ethnic minority groups [99] and a framework for coproduction of digital services for marginalized people living with complex and chronic conditions [100]. A number of papers have put forward design and assessment tools for equity in digital health [61,64,101-103]. A review of tools for inclusivity and cultural sensitivity, coproduction approaches, and equitable design processes could identify practical steps that could be taken by developers to promote equity in digital health.

Future research should assess how the frameworks identified in this scoping review can be used and applied to different ethnic minority groups and in the management of other health conditions. The complex intersections of factors associated with health and other inequalities should also be considered. For example, in England, some ethnic groups are more likely to live in deprived areas [104], and deprivation is associated with increased mortality across all ethnic groups, including White ethnicity [105]. Application of appropriate frameworks for engagement, implementation, and evaluation can improve the reach of measures to address broader health inequalities and target all underserved groups.

### Conclusions

Health inequalities continue to be a major focus in health policy and research globally. A number of frameworks have been put forward to address social determinants of health [15] or to improve inequalities in particular major chronic health conditions, such as cardiometabolic diseases [106]. As digital health approaches are encouraged and become more commonplace, we should use our existing theoretical understanding of the interaction between digital health approaches and health inequalities to improve equitable distribution of benefits, including to ethnic minority populations. We have produced a visual guide (Figure 2) to shape action when considering preventable or manageable chronic disease in the community that shows ethnic inequalities in outcomes, such as cardiometabolic disease.

## Appendix

Multimedia Appendix 1 PRISMA-ScR checklist.

Multimedia Appendix 2 Inclusion and exclusion criteria for literature searches.

Multimedia Appendix 3 Search strategy as used for SCOPUS.

Multimedia Appendix 4 Inclusion and exclusion guide for title and abstract screening.

Multimedia Appendix 5 Additional frameworks identified through abstract screening.

Multimedia Appendix 6 Data-charting form.

Multimedia Appendix 7 Summary of papers included in the data charting.

## Abbreviations

mHealth: mobile health
NHS: National Health Service
NIHR: National Institute for Health Research
PRISMA-ScR: Preferred Reporting Items for Systematic Reviews and Meta-Analyses for Scoping Reviews
RESET: relevance, evidence base, stages of intervention, ethnicity and trends

## References

[1] UK Ethnicity facts and figures. Population of England and Wales.

[2] Goff LM (2019). Ethnicity and type 2 diabetes in the UK. Diabet Med.

[3] Chaturvedi N (2003). Ethnic differences in cardiovascular disease. Heart.

[4] Murray E, Hekler E, Andersson G, Collins L, Doherty A, Hollis C, Rivera D, West R, Wyatt J (2016). Evaluating digital health interventions: key questions and approaches. Am J Prev Med.

[5] Beishuizen CRL, Stephan BCM, van Gool Willem A, Brayne C, Peters RJG, Andrieu S, Kivipelto M, Soininen H, Busschers WB, Moll van Charante Eric P, Richard E (2016). Web-based interventions targeting cardiovascular risk factors in middle-aged and older people: a systematic review and meta-analysis. J Med Internet Res.

[6] McLean G, Band R, Saunderson K, Hanlon P, Murray E, Little P, McManus Richard J, Yardley L, Mair F, DIPSS co-investigators (2016). Digital interventions to promote self-management in adults with hypertension systematic review and meta-analysis. J Hypertens.

[7] Pal K, Eastwood SV, Michie Susan, Farmer Andrew J, Barnard Maria L, Peacock Richard, Wood Bindie, Inniss Joni D, Murray Elizabeth (2013). Computer-based diabetes self-management interventions for adults with type 2 diabetes mellitus. Cochrane Database Syst Rev.

[8] Ramasawmy M, Poole L, Banerjee A (2021). Learning our lesson: using past policies to improve digital and ethnic inequalities beyond the pandemic. Arch Public Health.

[9] Captieux M, Pearce G, Parke HL, Epiphaniou E, Wild S, Taylor SJC, Pinnock H (2018). Supported self-management for people with type 2 diabetes: a meta-review of quantitative systematic reviews. BMJ Open.

[10] McAuley A (2014). Digital health interventions: widening access or widening inequalities?. Public Health.

[11] Chaturvedi N, Fuller J (1996). Ethnic differences in mortality from cardiovascular disease in the UK: do they persist in people with diabetes?. J Epidemiol Community Health.

[12] Holden RJ, Karsh B (2010). The technology acceptance model: its past and its future in health care. J Biomed Inform.

[13] Gücin Nö, Berk ÖS (2015). Technology acceptance in health care: an integrative review of predictive factors and intervention programs. Proc Soc Behav Sci.

[14] Greenhalgh T, Wherton J, Papoutsi C, Lynch J, Hughes G, A'Court Christine, Hinder S, Fahy N, Procter R, Shaw S (2017). Beyond adoption: a new framework for theorizing and evaluating nonadoption, abandonment, and challenges to the scale-up, spread, and sustainability of health and care technologies. J Med Internet Res.

[15] Solar O, Irwin A (2010). A conceptual framework for action on the social determinants of health. World Health Organization.

[16] Nadal C, Sas C, Doherty G (2020). Technology acceptance in mobile health: Scoping review of definitions, models, and measurement. J Med Internet Res.

[17] Rabin B, Brownson R, Haire-Joshu D, Kreuter M, Weaver N (2008). A glossary for dissemination and implementation research in health. J Public Health Manag Pract.

[18] OpenGrey.

[19] Grey literature report. New York Academy of Medicine.

[20] Tricco A, Lillie E, Zarin W, O'Brien Kelly K, Colquhoun H, Levac D, Moher D, Peters M, Horsley T, Weeks L, Hempel S, Akl E, Chang C, McGowan J, Stewart L, Hartling L, Aldcroft A, Wilson M, Garritty C, Lewin S, Godfrey C, Macdonald Marilyn T, Langlois EV, Soares-Weiser Karla, Moriarty Jo, Clifford Tammy, Tunçalp Özge, Straus Sharon E (2018). PRISMA extension for scoping reviews (PRISMA-ScR): checklist and explanation. Ann Intern Med.

[21] Ahmad A, Rasul T, Yousaf A, Zaman U (2020). Understanding factors influencing elderly diabetic patients’ continuance intention to use digital health wearables: extending the technology acceptance model (TAM). J Open Innov Technol Mark Complex.

[22] An J (2006). Theory development in health care informatics: information and communication technology acceptance model (ICTAM) improves the explanatory and predictive power of technology acceptance models. Stud Health Technol Inform.

[23] Campbell J, Aturinda I, Mwesigwa E, Burns B, Santorino D, Haberer J, Bangsberg D, Holden R, Ware N, Siedner M (2017). The technology acceptance model for resource-limited settings (TAM-RLS): a novel framework for mobile health interventions targeted to low-literacy end-users in resource-limited settings. AIDS Behav.

[24] Devito Dabbs Annette, Song M, Hawkins R, Aubrecht J, Kovach K, Terhorst L, Connolly M, McNulty M, Callan J (2011). An intervention fidelity framework for technology-based behavioral interventions. Nurs Res.

[25] Guttman N, Lev E, Segev E, Ayecheh S, Ziv L, Gadamo F, Dayan N, Yavetz G (2017). “I never thought I could get health information from the internet!”: unexpected uses of an internet website designed to enable Ethiopian immigrants with low/no literacy skills to browse health information. New Media Soc.

[26] Hossain N, Yokota F, Sultana N, Ahmed A (2019). Factors influencing rural end-users' acceptance of e-health in developing countries: a study on portable health clinic in Bangladesh. Telemed J E Health.

[27] Jacob C, Sanchez-Vazquez A, Ivory C (2020). Understanding clinicians' adoption of mobile health tools: a qualitative review of the most used frameworks. JMIR Mhealth Uhealth.

[28] Kamal SA, Shafiq M, Kakria P (2020). Investigating acceptance of telemedicine services through an extended technology acceptance model (TAM). Technol Soc.

[29] Kim J, Park H (2012). Development of a health information technology acceptance model using consumers' health behavior intention. J Med Internet Res.

[30] Li Q (2020). Healthcare at your fingertips: the acceptance and adoption of mobile medical treatment services among Chinese users. Int J Environ Res Public Health.

[31] Ondiege B, Clarke M (2017). Investigating user identification in remote patient monitoring devices. Bioengineering (Basel).

[32] Opoku D, Stephani V, Quentin W (2017). A realist review of mobile phone-based health interventions for non-communicable disease management in sub-Saharan Africa. BMC Med.

[33] Putri KYS, Abdullah Z, Istiyanto SB, Anumudu CE (2020). The antecedents and consequences of e-health literacy in the pharmaceutical industry: An agenda for future research. Int J App Pharm.

[34] Su Y, Huang S, Wu Y, Chen C (2020). Factors affecting patients' acceptance of and satisfaction with cloud-based telehealth for chronic disease management: a case study in the workplace. Appl Clin Inform.

[35] Venkatesh V, Morris M, Davis G, Davis F (2003). User acceptance of information technology: toward a unified view. MIS Q.

[36] Zhou M, Zhao L, Kong N, Campy KS, Qu S, Wang S (2019). Factors influencing behavior intentions to telehealth by Chinese elderly: an extended TAM model. Int J Med Inform.

[37] Bettiga D, Lamberti L, Lettieri E (2020). Individuals' adoption of smart technologies for preventive health care: a structural equation modeling approach. Health Care Manag Sci.

[38] Alaiad A, Alsharo M, Alnsour Y (2019). The determinants of m-health adoption in developing countries: an empirical investigation. Appl Clin Inform.

[39] Arfi WB, Nasr IB, Kondrateva G, Hikkerova L (2021). The role of trust in intention to use the IoT in eHealth: application of the modified UTAUT in a consumer context. Technol Forecast Soc Change.

[40] Chang I, Hsu H (2012). Predicting medical staff intention to use an online reporting system with modified unified theory of acceptance and use of technology. Telemed J E Health.

[41] Hoque R, Sorwar G (2017). Understanding factors influencing the adoption of mHealth by the elderly: an extension of the UTAUT model. Int J Med Inform.

[42] Sari H, Othman M, Al-Ghaili A, Saeed F, Gazem N, Mohammed F, Busalim A (2019). A proposed conceptual framework for mobile health technology adoption among employees at workplaces in Malaysia. Recent Trends in Data Science and Soft Computing. Advances in Intelligent Systems and Computing.

[43] Tavares J, Oliveira T (2016). Electronic health record patient portal adoption by health care consumers: an acceptance model and survey. J Med Internet Res.

[44] Atun R, de Jongh T, Secci F, Ohiri K, Adeyi O (2010). Integration of targeted health interventions into health systems: a conceptual framework for analysis. Health Policy Plan.

[45] Zhao Y, Ni Q, Zhou R (2018). What factors influence the mobile health service adoption? a meta-analysis and the moderating role of age. Int J Inf Manage.

[46] Despont-Gros C, Fabry P, Muller H, Geissbuhler A, Lovis C (2004). User acceptance of clinical information systems: a methodological approach to identify the key dimensions allowing a reliable evaluation framework. Stud Health Technol Inform.

[47] Holden RJ, Karsh B (2009). A theoretical model of health information technology usage behaviour with implications for patient safety. Behav Inf Technol.

[48] Aljarullah A, Crowder R, Wills G (2017). A framework for the adoption of EHRs by primary healthcare physicians in the kingdom of Saudi Arabia. https://ieeexplore.ieee.org/document/8354670.

[49] Chang H (2015). Evaluation framework for telemedicine using the logical framework approach and a fishbone diagram. Healthc Inform Res.

[50] Fanta G, Pretorius L, Erasmus L (2016). A system dynamics model of ehealth acceptance: a sociotechnical perspective.

[51] Lowe B, Fraser I, Souza-Monteiro DM (2015). A change for the better? digital health technologies and changing food consumption behaviors. Psychol Mark.

[52] Zhang C, Lakens D, IJsselsteijn W (2021). Theory integration for lifestyle behavior change in the digital age: an adaptive decision-making framework. J Med Internet Res.

[53] Damschroder L, Aron D, Keith R, Kirsh S, Alexander J, Lowery J (2009). Fostering implementation of health services research findings into practice: a consolidated framework for advancing implementation science. Implement Sci.

[54] Salisbury C, Thomas C, O'Cathain Alicia, Rogers A, Pope C, Yardley L, Hollinghurst S, Fahey T, Lewis G, Large S, Edwards L, Rowsell A, Segar J, Brownsell S, Montgomery A (2015). Telehealth in chronic disease: mixed-methods study to develop the tech conceptual model for intervention design and evaluation. BMJ Open.

[55] Glasgow RE, Vogt TM, Boles SM (1999). Evaluating the public health impact of health promotion interventions: the RE-AIM framework. Am J Public Health.

[56] Khatun F, Heywood AE, Ray PK, Hanifi S, Bhuiya A, Liaw S (2015). Determinants of readiness to adopt mHealth in a rural community of Bangladesh. Int J Med Inform.

[57] Addotey-Delove M, Scott RE, Mars M (2020). Review of patients’ perspectives of m-health adoption factors in the developing world. development of a proposed conceptual framework. Informatics Med Unlocked.

[58] Puuronen S, Vasilyeva E, Pechenizkiy M, Tesanovic A (2010). A holistic framework for understanding acceptance of Remote Patient Management (RPM) systems by non-professional users. Proceedings of the 23rd International Symposium on Computer-Based Medical Systems.

[59] Perski O, Blandford A, West R, Michie S (2017). Conceptualising engagement with digital behaviour change interventions: a systematic review using principles from critical interpretive synthesis. Transl Behav Med.

[60] O'Connor Siobhan, Hanlon P, O'Donnell Catherine A, Garcia S, Glanville J, Mair F (2016). Understanding factors affecting patient and public engagement and recruitment to digital health interventions: a systematic review of qualitative studies. BMC Med Inform Decis Mak.

[61] Latulippe K, Hamel C, Giroux D (2017). Social health inequalities and eHealth: a literature review with qualitative synthesis of theoretical and empirical studies. J Med Internet Res.

[62] Leach MJ, Segal L (2011). Patient attributes warranting consideration in clinical practice guidelines, health workforce planning and policy. BMC Health Serv Res.

[63] Bodie GD, Dutta MJ (2008). Understanding health literacy for strategic health marketing: eHealth literacy, health disparities, and the digital divide. Health Mark Q.

[64] Foley K, Freeman T, Ward P, Lawler A, Osborne R, Fisher M (2021). Exploring access to, use of and benefits from population-oriented digital health services in Australia. Health Promot Int.

[65] Schillinger D (2020). The intersections between social determinants of health, health literacy, and health disparities. Stud Health Technol Inform.

[66] Wilkinson E, Waqar M, Sinclair A, Randhawa G (2016). Meeting the challenge of diabetes in ageing and diverse populations: a review of the literature from the UK. J Diabetes Res.

[67] Campbell DJT, Manns BJ, Leblanc P, Hemmelgarn BR, Sanmartin C, King-Shier K (2016). Finding resiliency in the face of financial barriers: development of a conceptual framework for people with cardiovascular-related chronic disease. Medicine (Baltimore).

[68] Wildenbos GA, Peute L, Jaspers M (2018). Aging barriers influencing mobile health usability for older adults: a literature based framework (MOLD-US). Int J Med Inform.

[69] An J, Hayman L, Panniers T, Carty B (2007). Theory development in nursing and healthcare informatics: a model explaining and predicting information and communication technology acceptance by healthcare consumers. ANS Adv Nurs Sci.

[70] Dam L, Roy D, Atkin DJ, Rogers D (2018). Applying an integrative technology adoption paradigm to health app adoption and use. J Broadcast Electron Media.

[71] Kujala S, Ammenwerth E, Kolanen H, Ervast M (2020). Applying and extending the FITT framework to identify the challenges and opportunities of successful ehealth services for patient self-management: qualitative interview study. J Med Internet Res.

[72] Szinay D, Perski O, Jones A, Chadborn T, Brown J, Naughton F (2021). Perceptions of factors influencing engagement with health and wellbeing apps: a qualitative study using the COM-B model and Theoretical Domains Framework. Qeios.

[73] Yang Meier Dong, Barthelmess P, Sun W, Liberatore F (2020). Wearable technology acceptance in health care based on national culture differences: cross-country analysis between Chinese and Swiss consumers. J Med Internet Res.

[74] Antonio MG, Petrovskaya O (2019). Towards developing an eHealth equity conceptual framework. Stud Health Technol Inform.

[75] Crawford A, Serhal E (2020). Digital health equity and COVID-19: the innovation curve cannot reinforce the social gradient of health. J Med Internet Res.

[76] Jack L, Toston T, Jack NH, Sims M (2010). A gender-centered ecological framework targeting Black men living with diabetes: integrating a "masculinity" perspective in diabetes management and education research. Am J Mens Health.

[77] Jenkins C, Pope C, Magwood G, Vandemark L, Thomas V, Hill K, Linnen F, Beck LS, Zapka J (2010). Expanding the chronic care framework to improve diabetes management: the REACH case study. Prog Community Health Partnersh.

[78] Dover D, Belon A (2019). The health equity measurement framework: a comprehensive model to measure social inequities in health. Int J Equity Health.

[79] Dutta-Bergman M, Murero M, Rice R (2006). Media use theory and internet use for health care. The Internet and Health Care.

[80] Davis FD (1989). Perceived usefulness, perceived ease of use, and user acceptance of information technology. MIS Q.

[81] Marmot M, Bell R (2019). Social determinants and non-communicable diseases: time for integrated action. BMJ.

[82] (2022). Ethnic inequalities in healthcare: a rapid evidence review. NHS Race and Health Observatory.

[83] Armaou M, Araviaki E, Musikanski L (2019). eHealth and mhealth interventions for ethnic minority and historically underserved populations in developed countries: an umbrella review. Int J Com WB.

[84] Lawton J, Ahmad N, Hanna L, Douglas M, Hallowell N (2006). 'I can't do any serious exercise': barriers to physical activity amongst people of Pakistani and Indian origin with Type 2 diabetes. Health Educ Res.

[85] Sriskantharajah J, Kai J (2007). Promoting physical activity among South Asian women with coronary heart disease and diabetes: what might help?. Fam Pract.

[86] Veinot T, Ancker J, Bakken S (2019). Health informatics and health equity: improving our reach and impact. J Am Med Inform Assoc.

[87] Antonio M, Petrovskaya O, Lau F (2019). Is research on patient portals attuned to health equity? a scoping review. J Am Med Inform Assoc.

[88] Grossman LV, Masterson Creber RM, Benda NC, Wright D, Vawdrey DK, Ancker JS (2019). Interventions to increase patient portal use in vulnerable populations: a systematic review. J Am Med Inform Assoc.

[89] Shaw J, Brewer LC, Veinot T (2021). Recommendations for health equity and virtual care arising from the COVID-19 pandemic: narrative review. JMIR Form Res.

[90] Greenhalgh T, Rosen R, Shaw SE, Byng R, Faulkner S, Finlay T, Grundy E, Husain L, Hughes G, Leone C, Moore L, Papoutsi C, Pope C, Rybczynska-Bunt S, Rushforth A, Wherton J, Wieringa S, Wood GW (2021). Planning and evaluating remote consultation services: a new conceptual framework incorporating complexity and practical ethics. Front Digit Health.

[91] (2019). NHS long term plan. National Health Service.

[92] NHS diabetes prevention programme – digital stream. NHS England.

[93] Cardiology digital playbook. NHS Transformation Directorate.

[94] Robbins T, Hudson S, Ray P, Sankar S, Patel K, Randeva H, Arvanitis T (2020). COVID-19: a new digital dawn?. Digit Health.

[95] Digital transformation. National Health Service.

[96] Honeyman M, Maguire D, Evans H, Davies A (2020). Digital technology and health inequalities: a scoping review. Public Health Wales NHS Trust.

[97] Gough D, Thomas J, Oliver S (2012). Clarifying differences between review designs and methods. Syst Rev.

[98] Arksey H, O'Malley L (2005). Scoping studies: towards a methodological framework. Int J Soc Res Methodol Theory Pract.

[99] Liu J, Davidson E, Bhopal R, White M, Johnson M, Netto G, Deverill M, Sheikh A (2012). Adapting health promotion interventions to meet the needs of ethnic minority groups: mixed-methods evidence synthesis. Health Technol Assess.

[100] Kayser L, Nøhr C, Bertelsen P, Botin L, Villumsen S, Showell C, Turner P (2018). Theory and practice in digital behaviour change: a matrix framework for the co-production of digital services that engage, empower and emancipate marginalised people living with complex and chronic conditions. Informatics.

[101] Mobasseri K, Azami-Aghdash S, Khanijahani A, Khodayari-Zarnaq R (2020). The main issues and challenges older adults face in the sars-cov-2 pandemic: a scoping review of literature. Iran J Public Health.

[102] Benkhalti M, Espinoza M, Cookson R, Welch V, Tugwell P, Dagenais P (2021). Development of a checklist to guide equity considerations in health technology assessment. Int J Technol Assess Health Care.

[103] Samuels-Kalow M, Jaffe T, Zachrison K (2021). Digital disparities: designing telemedicine systems with a health equity aim. Emerg Med J.

[104] (2017). Public health outcomes framework: health equity report. focus on ethnicity. Public Health England.

[105] Wan Y, Robbins A, Apea V, Orkin C, Pearse R, Puthucheary Z, Prowle J (2021). Ethnicity and acute hospital admissions: multi-center analysis of routine hospital data. EClinicalMedicine.

[106] Mensah GA, Dunbar SB (2006). A framework for addressing disparities in cardiovascular health. J Cardiovasc Nurs.

