# Supplementary material for: Frameworks for Implementation, Uptake, and Use of Cardiometabolic Disease–Related Digital Health Interventions in Ethnic Minority Populations: Scoping Review
Source: JMIR Cardio. 2022 Aug 11;6(2):e37360. doi: 10.2196/37360 (PMC9412726; doi:10.2196/37360)
Supplement: Multimedia Appendix 2 [file cardio_v6i2e37360_app2.docx]

Appendix 2. Inclusion and exclusion criteria for literature searches.

| **Inclusion criteria** | |
| --- | --- |
| Source type | Published articles, grey literature |
| Article topic | Framework (model, theory, taxonomy, etc.) for adoption and acceptance of intervention |
| Intervention | Digital health and/or cardiometabolic disease |
| Population | All populations; populations experiencing health inequalities; South Asian populations |
| **Exclusion criteria** | |
| Articles which describe or evaluate effectiveness of DHIs in cardiometabolic disease, but which do not put forward a framework or model for understanding | |
| Non-English articles | |
