# Supplementary material for: Frameworks for Implementation, Uptake, and Use of Cardiometabolic Disease–Related Digital Health Interventions in Ethnic Minority Populations: Scoping Review
Source: JMIR Cardio. 2022 Aug 11;6(2):e37360. doi: 10.2196/37360 (PMC9412726; doi:10.2196/37360)
Supplement: Multimedia Appendix 3 [file cardio_v6i2e37360_app3.docx]

Appendix 3. Search strategy as used for SCOPUS.

| Search Strategy - SCOPUS |
| --- |
| *Frameworks for digital health adoption*   - TITLE-ABS-KEY ( "digital health" AND framework AND adopt* ) - TITLE-ABS-KEY ( "digital health" AND ( framework OR model OR theor* OR taxon* ) AND adopt* ) - TITLE-ABS-KEY ( "digital health"  AND  framework  AND  ( accept*  OR  appl*  OR  roll-out  OR  uptake  OR  implement ) ) - TITLE-ABS-KEY ( "digital health"  AND  model  AND  ( accept*  OR  appl*  OR  roll-out  OR  uptake  OR  implement ) ) - TITLE-ABS-KEY ( ( "digital health"  OR  "health tech*"  OR  e- medicine  OR  emedicine  OR  ehealth  OR  e-health  OR  telemedicine )  AND  framework  AND  accept* ) |
| *Frameworks for health inequalities in access or implementation of digital health*   - TITLE-ABS-KEY ( "digital health"  AND  framework  AND  adop*  AND  "South Asian" ) - TITLE-ABS-KEY ( "digital health"  AND  framework  AND  access*  AND  "South Asian" ) - TITLE-ABS-KEY ( "digital health" AND access* AND "South Asian" ) - TITLE-ABS-KEY ( "digital health" AND adopt* AND "South Asian" ) - TITLE-ABS-KEY ( "digital health" AND framework AND "South Asian" ) - TITLE-ABS-KEY ( "digital health" AND model AND "South Asian" ) - *Above repeated with “black”; “African American”, “Hispanic”, “latino”, “indigenous”, “Asian”, “Chinese”)* - TITLE-ABS-KEY ( "digital health" AND access* AND ethnic* ) - TITLE-ABS-KEY ( "digital health"  AND  adopt*  AND  ethnic* ) - TITLE-ABS-KEY ( "digital health"  AND  framework  AND  ethnic* ) - TITLE-ABS-KEY ( "digital health"  AND  model  AND  ethnic* ) - *Above four repeated with “race”, “racial*” and “racis*”)* - TITLE-ABS-KEY ( "digital health" AND "South Asian" ) - TITLE-ABS-KEY ( "health tech*" AND "South Asian" ) - TITLE-ABS-KEY ( ( telemedicine OR e-medicine OR emedicine OR ehealth OR e-health ) AND "South Asian" ) - *Above three repeated with “black”; “African American”, “Hispanic”, “latino”, “indigenous”, “Asian”, “Chinese”, “race”, “racial*” and “racis*”)* - TITLE-ABS-KEY ( ( "digital health" OR "health tech*" OR telemedicine OR e-medicine OR emedicine OR ehealth OR e-health ) AND framework AND inequal* ) - TITLE-ABS-KEY ( ( "digital health" OR "health tech*" OR telemedicine OR e-medicine OR emedicine OR ehealth OR e-health ) AND model AND inequal* ) - TITLE-ABS-KEY ( ( "digital health" OR "health tech*" OR telemedicine OR e-medicine OR emedicine OR ehealth OR e-health ) AND theory AND inequal* ) - *Above three repeated with disparit* replacing inequal** - TITLE-ABS-KEY ( ( "digital health"  OR  "health tech*"  OR  telemedicine  OR  e-medicine  OR  emedicine  OR  ehealth  OR  e-health )  AND  framework  AND  discrim* ) - TITLE-ABS-KEY ( "digital divide" AND health AND ( framework OR model OR theor* OR taxon* ) ) - TITLE-ABS-KEY ( "digital health"  AND  disparit*  AND  ( framework  OR  model  OR  theor*  OR  taxon* ) ) |
| *Frameworks for adoption of interventions for cardiovascular disease or diabetes mellitus*   - TITLE-ABS-KEY ( cardiometabolic  AND  framework  AND  ( adopt*  OR  application  AND accept*  OR  roll-out  OR  uptake  OR  implement* ) ) - TITLE-ABS-KEY ( cardiometabolic  AND  adopt*  AND  ( framework  OR  model  OR  theor*  OR  taxon* ) ) - TITLE-ABS-KEY ( ( cardiometabolic  OR  cardiovascular  OR  diabet*  OR  "coronary heart disease"  OR  stroke  OR  "myocardial infarction"  OR  hypertension )  AND  intervention  AND  adopt*  AND  framework ) |
| *Frameworks for health inequalities of interventions for cardiovascular disease or diabetes mellitus*   - TITLE-ABS-KEY ( cardiometabolic  AND  adopt*  AND  framework  AND  ( inequal*  OR  "South Asian"  OR  ethnic*  OR  minorit*  OR  bme  OR  bame ) ) - TITLE-ABS-KEY ( cardiometabolic AND adopt* AND framework AND (black OR "african american" OR hispanic OR latino OR asian OR indigenous OR chinese) ) - TITLE-ABS-KEY ( cardiometabolic AND adopt* AND framework AND race ), repeated for racial* and racis* - *Repeated, replacing framework for “model” and “theory”* - TITLE-ABS-KEY ( cardiometabolic  AND  framework  AND  ( inequal*  OR  "South Asian"  OR  ethnic*  OR  minorit*  OR  bme  OR  bame ) ) - TITLE-ABS-KEY ( cardiometabolic  AND  framework  AND  (black OR "african american" OR hispanic OR latino OR asian OR indigenous OR chinese)) - TITLE-ABS-KEY ( ( cardiometabolic OR cardiovascular OR diabet* OR "coronary heart disease" OR stroke OR "myocardial infarction" OR hypertension ) AND framework AND inequal* ) - TITLE-ABS-KEY ( ( cardiometabolic OR cardiovascular OR diabet* OR "coronary heart disease" OR stroke OR "myocardial infarction" OR hypertension ) AND framework AND "digital divide" ) - TITLE-ABS-KEY ( ( cardiometabolic OR cardiovascular OR diabet* OR "coronary heart disease" OR stroke OR "myocardial infarction" OR hypertension ) AND framework AND disparit* ) |
