# Supplementary material for: Frameworks for Implementation, Uptake, and Use of Cardiometabolic Disease–Related Digital Health Interventions in Ethnic Minority Populations: Scoping Review
Source: JMIR Cardio. 2022 Aug 11;6(2):e37360. doi: 10.2196/37360 (PMC9412726; doi:10.2196/37360)
Supplement: Multimedia Appendix 4 [file cardio_v6i2e37360_app4.docx]

Appendix 4. Inclusion and exclusion criteria for title and abstract screening.

| Inclusion/Exclusion criteria | |
| --- | --- |
| Article type | Inclusion   - Proposes new framework - Updates or adapts existing framework   Exclusion   - Test existing framework against data without proposing updates - Other types of framework (e.g. delivery, research, public engagement) |
| Scope/scale of framework | *Definition*   - Framework should help us understand the factors that affect whether an innovation reaches the end-user (e.g. patient) and/or whether they choose to use it - A relevant framework could consider some or all steps of the pathway – from individual factors to population/policy |
| Framework subject | *Digital Health Intervention adoption/implementation/acceptance/etc.*   - Includes digital element that patients/public will interact with - Definitions can include e-health/e-medicine/telemedicine/health tech/m-health, or broader scope e.g. health innovations or health technology - Systems- or health care professional- focused innovation which patients or public will not interact with – e.g. new information systems   *Cardiometabolic disease*   - Consider factors affecting adoption/implementation of (any) interventions, or where CMD overlaps with health inequalities. - Describe or evaluate effectiveness of interventions in this population, without presenting new or adapted framework   *Health inequalities*   - Social determinants of health, health inequalities, ethnicity |

These expanded inclusion and exclusion criteria were developed to support decision making. Excluded frameworks focussed on: assessing national readiness for digital, delivery models of care, stakeholder engagement, health technology design process, and practical methods for evaluation; or were framed as being specific to the need of particular groups; or exclusively for health conditions other than cardiometabolic disease.
