# Supplementary material for: Frameworks for Implementation, Uptake, and Use of Cardiometabolic Disease–Related Digital Health Interventions in Ethnic Minority Populations: Scoping Review
Source: JMIR Cardio. 2022 Aug 11;6(2):e37360. doi: 10.2196/37360 (PMC9412726; doi:10.2196/37360)
Supplement: Multimedia Appendix 5 [file cardio_v6i2e37360_app5.docx]

Appendix 5. Frameworks identified through abstract screening.

| **Major frameworks commonly mentioned at abstract screening phase, sought and added to full text screening for further review.** |
| --- |
| - ADAPT-ITT framework (Wingood and Diclemente 2008) - Bandura model of health promotion (Bandura 1998) - Behavior Interventions using Technology (BIT) frameworks (Mohr et al. 2014) - Benefit Evaluation (BE) framework (Lau, Hagens, and Muttitt 2007) - Collective resources model (*see* Bansler 1989) - Competency Opportunity, Motivation Behaviour System (COM-B model) (Michie, van Stralen, and West 2011) - Consolidated Framework for Implementation Research (CFIR) (Damschroder et al. 2009) - Diffusion of innovations Theory and variations (Rogers 1995) - Effective Coverage framework (Shengelia et al. 2005) - Life course approach (Kuh and Shlomo, 1997) - Nonadoption, Abandonment, and Challenges to the Scale-up, Spread, and Suitability framework (Greenhalgh et al. 2017b) - PRECEDE-PROCEED model (Green, 2005) - Reach, Effectiveness, Adoption, Implementation, and Maintenance (RE-AIM) (Glasgow et al. 2019) - Social determinants of health framework (Solar and Irwin 2010) - Stakeholder Empowered Adoption Model (Marshall 2013) - Syndemic theory/framework (Singer, 1994) - System Usability Scale (Brooke, 1996) - Spell out in full (TAM) (and variants) (Davis, 1989) - Theory of Reasoned Action (Fishbein & Ajzen, 1975) - Theory of Planned Behaviour (Ajzen, 1991) - Transtheoretical model of behaviour change (Prochaska and DiClemente, 1983) - Unified Theory of Acceptance and Use of Technology (UTAUT) (Venkatesh et al. 2003) |
