# Supplementary material for: Frameworks for Implementation, Uptake, and Use of Cardiometabolic Disease–Related Digital Health Interventions in Ethnic Minority Populations: Scoping Review
Source: JMIR Cardio. 2022 Aug 11;6(2):e37360. doi: 10.2196/37360 (PMC9412726; doi:10.2196/37360)
Supplement: Multimedia Appendix 6 [file cardio_v6i2e37360_app6.docx]

Appendix 6. Data charting form

Citation

- Year
- Author(s)
- Title
- Journal, Volume (issues): pages / other
- Country of first author affiliation

Source details

- Source type (published, preprint, conference abstract, policy, other)
- Article type (review and synthesis, review and commentary, original research, other)

Framework details

- Framework type (new framework, review of existing framework, adaptation of existing framework)
- Framework name
- If adapted, original framework(s), author
- Describe purpose/scope of framework/adaptation
- Describe how framework was tested
- If general review, describe key focus

Scope

- Context (population)
- Component(s) (DHI, cardiometabolic, health inequalities)
- Domains (individual, intervention, healthcare system, society)
- Lens (who benefits from the framework) (patient, clinician, technology, provider/healthcare system)

Health inequalities

- Framework incorporates health inequalities?

Image

Additional comments
