# Supplementary material for: Frameworks for Implementation, Uptake, and Use of Cardiometabolic Disease–Related Digital Health Interventions in Ethnic Minority Populations: Scoping Review
Source: JMIR Cardio. 2022 Aug 11;6(2):e37360. doi: 10.2196/37360 (PMC9412726; doi:10.2196/37360)
Supplement: Multimedia Appendix 7 [file cardio_v6i2e37360_app7.docx]

Appendix 7. Summary of papers included in the data charting.

| Author(s) (Year) | Title | Framework type | Framework name | Context | Component (DHI / CMD /inequalities) | Framework purpose |
| --- | --- | --- | --- | --- | --- | --- |
| Addotey-Delove (2020) | Review of patients’ perspectives of m-health adoption factors in the developing world. Development of a proposed conceptual framework | New framework | Conceptual framework for increased patient adoption of m-health by patients in the developing world | “Developing world” (as defined by paper) | DHIs, health inequalities | Key variables that need to be addressed to guide implementation of m-health adoption by patients in developing world |
| Ahmad et al (2020) | Understanding Factors Influencing Elderly Diabetic Patients’ Continuance Intention to Use Digital Health Wearables: Extending the Technology Acceptance Model (TAM) | Adaptation of existing framework(s) | Conceptual model of continuance intention | Individuals aged over 60, with diabetes, living in Bangladesh | DHIs, cardiometabolic diseases | Predicting use of digital wearables in elderly patients with diabetes in Bangladesh |
| Alaiad et al (2019) | The Determinants of M-Health Adoption in Developing Countries: An Empirical Investigation | Adaptation of existing framework(s) | No name specified | Younger people in Jordan | DHIs | Facilitators and barriers for adoption of mhealth in developing countries |
| Aljarullah et al (2018) | A framework for the adoption of EHRs by primary healthcare physicians in the kingdom of Saudi Arabia | New framework | Framework for the Acceptance of EHRs by Primary Healthcare Physicians in the KSA | Health care professionals in Saudi Arabia | DHIs | Identification of factors that are likely to influence the adoption of EHRs by primary healthcare physicians in Saudi Arabia |
| An et al. (2007) | Theory development in nursing and healthcare informatics: A model explaining and predicting information and communication technology acceptance by healthcare consumers | Adaptation of existing framework(s) | Information and Communication Technology Acceptance Model (ICTAM) | Users of health information on the internet | DHIs | Integrating multidisciplinary perspectives from divergent frameworks and empirical findings into a unified model with regard to healthcare consumers' acceptance and usage behaviour of information and services on the Internet. |
| Antonio et al (2019) | Towards developing an eHealth equity conceptual framework | Adaptation of existing framework(s) | eHealth Equity Framework | eHealth users | DHIs, health inequalities | Adapting the CSDH to bring together the concepts of health equity, social determinants of health inequities and ehealth. |
| Arfi et al (2021) | The role of trust in intention to use the IoT in eHealth: Application of the modified UTAUT in a consumer context | Adaptation of existing framework(s) | Framework Model of the determinants of behavioural intentions relating to IoT healthcare devices | France | DHIs | Examine factors influencing patients’ adoption of the internet of things for eHealth, with particular consideration to the trust-risk relationship |
| Atun et al (2009) | Integration of targeted health interventions into health systems: A conceptual framework for analysis | New framework | Conceptual framework for analysing integration of targeted health interventions into health systems. | DHIs | DHIs | Examine how health interventions are integrated into health systems |
| Bettiga et al (2020) | Individuals’ adoption of smart technologies for preventive health care: a structural equation modeling approach | Adaptation of existing framework(s) | No name specified | Non-hypertensive individuals, Italy | DHIs, cardiometabolic disease | Explore the determinants of users’ willingness to adopt and pay for a mobile health care application for cardiovascular prevention |
| Bodie et al (2008) | Understanding Health Literacy for Strategic Health Marketing: eHealth Literacy, Health Disparities, and the Digital Divide | New framework | The Updated Integrative Model of eHealth Use | US - general population | DHIs , health inequalities | Integrates ehealth access and use into a model of social and health disparities. |
| Campbell et al (2016) | Finding resiliency in the face of financial barriers Development of a conceptual framework for people with cardiovascular-related chronic disease | New framework | Conceptual framework for understanding the development and role of financial barriers for patients with cardiovascular-related chronic diseases | Canada; patients with cardiovascular-related chronic disease | DHIs; health inequalities; cardiometabolic disease | Maps development and role of financial barriers to patient outcomes for patients with cardiovascular-related chronic disease |
| Campbell et al (2017) | The Technology Acceptance Model for Resource-Limited Settings (TAM-RLS): A Novel Framework for Mobile Health Interventions Targeted to Low-Literacy End-Users in Resource-Limited Settings | Adaptation of existing framework(s) | Technology Acceptance Model for Resource-Limited Settings (TAM-RLS) | people living with HIV in rural Uganda | DHIs and health inequalities | model of mHealth technology acceptance based upon end-user experiences in rural Uganda |
| Chang (2015) | Evaluation Framework for Telemedicine Using the Logical Framework Approach and a Fishbone Diagram | New framework | No name specified | DHIs (telehealth implementation); no specific population | DHIs (telehealth implementation) | Part a) provides evaluation steps for implementation of telehealth; part b) provides detail on categories for factors and barriers of telemedicine implementation |
| Chang and Hsu (2012) | Predicting Medical Staff Intention to Use an Online Reporting System with Modified Unified Theory of Acceptance and Use of Technology | Adaptation of existing framework(s) | No name specified | Taiwan; HCP based at medical centres and regional hospitals | DHIs | Model to understand intention of medical staff to use patient safety record system |
| Crawford et al (2020) | Digital health equity and COVID-19: The innovation curve cannot reinforce the social gradient of health | Adaptation of existing framework(s) | The Digital Health Equity Framework (DHEF) | not specified (users of DHIs) | Health inequalities (ethnicity included as 'cultural contexts') and DHIs | to identify the digital determinants of health and their links to digital health equity. |
| Dam et al (2018) | Applying an Integrative Technology Adoption Paradigm to Health App Adoption and Use | Adaptation of existing framework(s) | Integrated Technology Adoption Model | US - undergraduate students ('digital native' cohort) | DHIs | Considers adoption and use in context of micro- to macro- social factors (from interpersonal to mass communication) |
| Damschroder et al (2009) | Fostering implementation of health services research findings into practice: a consolidated framework for advancing implementation science | New framework | Consolidated Framework For Implementation Research (CFIR) | General; implementation of interventions in health | Adoption/implementation | Mapping of characteristics that influence implementation effectiveness |
| Despont-Gros et al (2004) | User acceptance of Clinical Information Systems: A methodological approach to identify the key dimensions allowing a reliable evaluation framework | New framework | No name specified | Clinical information systems | DHIs | Uptake of clinical information systems in health settings |
| DeVito Dabbs et al (2011) | An Intervention Fidelity Framework for Technology-Based Behavioral Interventions | Adaptation of existing framework(s) | Intervention fidelity framework for technology-based behavioral interventions | DHIs (intervention fidelity) | DHIs (intervention fidelity) | Model places TAM in context of wider process related to intervention fidelity including delivery and receipt |
| Fanta et al (2016) | A System Dynamics Model of eHealth Acceptance: A Sociotechnical Perspective | New framework | Sociotechnical framework of technology acceptance | General | DHIs | Ehealth acceptance taking into account technological dimensions |
| Foley et al (2020) | Exploring access to, use of and benefits from population-oriented digital health services in Australia | New framework | No name specified | Australia (high and low socioeconomic areas) | DHIs; health inequalities (not specifically ethnicity but has 'social and economic resources') | Understanding factors influencing digital health service outcomes |
| Glasgow et al (1999) | Evaluating the Public Health Impact of Health Promotion Interventions: The RE-AIM Framework | New framework | The RE-AIM Framework | Public health interventions, general | (Public health) intervention | Evaluate (factors affecting) public health impact of an intervention |
| Greenhalgh et al (2017) | Beyond Adoption: A New Framework for Theorizing and Evaluating Nonadoption, Abandonment, and Challenges to the Scale-Up, Spread, and Sustainability of Health and Care Technologies | New framework | nonadoption, abandonment, scale-up, spread, and sustainability (NASSS) | General (although developed in UK) | DHIs (health and care technologies). included socio-cultural influences (not ethnicity explicitly) | Predict and evaluate success of technology-supported health or social care program |
| Guttman et al (2017) | I never thought I could get health information from the Internet!”: Unexpected uses of an Internet website designed to enable Ethiopian immigrants with low/no literacy skills to browse health information | Adaptation of existing framework(s) | Expanded culture centred Technology Acceptance model | Israeli Ethiopian immigrants | Health inequalities/DHIs (not ethnicity but cultural identity) | Ehealth use amongst Israeli Ethiopian immigrants with low literacy skills |
| Holden and Karsh (2012) | The Technology Acceptance Model: Its past and its future in health care | Review of frameworks | Technology Acceptance Model | n/a | n/a | n/a |
| Hoque et al (2017) | Understanding factors influencing the adoption of mHealth by the elderly: An extension of the UTAUT model | Adaptation of existing framework(s) | No name specified | Bangladesh, older adults living in Dhaka City Corporation | DHIs (mhealth) | To apply it to elderly users’ intention to adopt and use mHealth services |
| Hossain et al (2019) | Factors Influencing Rural End-Users' Acceptance of e-Health in Developing Countries: A Study on Portable Health Clinic in Bangladesh | Adaptation of existing framework(s) | No name specified | Bangladesh (rural) | DHIs | Combined variables from existing frameworks, and inclusion of consumer behaviour (e.g. cost, privacy, advertisement) |
| Jack et al (2008) | A Gender-Centered Ecological Framework Targeting Black Men Living With Diabetes: Integrating a “Masculinity” Perspective in Diabetes Management and Education Research | Adaptation of existing framework(s) | A gender-centered diabetes management education ecological framework | Black men with diabetes, US | Cardiometabolic disease (diabetes); Health inequalities, ethnicity (included as a demographic factor) | Pathways through which diabetes-related health disparities are influenced |
| Jacob et al (2020) | Understanding Clinicians’ Adoption of Mobile Health Tools: A Qualitative Review of the Most Used Frameworks | Adaptation of existing framework(s) | Consolidated framework of the factors impacting clinicians adaptation of mobile health | Clinicians | DHIs | Clinicians use of Mhealth |
| Jenkins et al (2010) | Expanding the Chronic Care Framework to Improve Diabetes Management: The REACH Case Study | Adaptation of existing framework(s) | Community Chronic Care Model | US - Charleston and Georgetown; Community management of chronic care needs | Health inequalities; Diabetes | Extending the CCM to include a community focus for improving diabetes self-management and reducing health disparities. |
| Kamal et al (2020) | Investigating acceptance of telemedicine services through an extended technology acceptance model (TAM) | Adaptation of existing framework(s) | No name provided | Rural Pakistan | DHIs (telemedicine) | To examine the factors influencing the acceptance of telemedicine services among the rural population of Pakistan. |
| Khatun et al (2015) | Determinants of readiness to adopt mHealth in a rural community of Bangladesh | New framework | mhealth readiness | Bangladesh (rural) | DHIs | to describe the influence of community readiness for mHealth |
| Kim and Park (2012) | Development of a Health Information Technology Acceptance Model Using Consumers’ Health Behavior Intention | Adaptation of existing framework(s) | Health Information Technology Acceptance Model (HITAM). | South Korea | DHIs (Health Information Technology) | Extend TAM to consider health consumer's behavioural intention of using health information technology |
| Kujala et al (2020) | Applying and Extending the FITT Framework to Identify the Challenges and Opportunities of Successful eHealth Services for Patient Self-Management: Qualitative Interview Study | Adaptation of existing framework(s) | Extended FITT | DHI - General; research carried out in Finland | self-management ehealth services | To include patients as an additional dimension to the existing framework |
| Latulippe et al (2017) | Social Health Inequalities and eHealth: A Literature Review With Qualitative Synthesis of Theoretical and Empirical Studies | New framework | Not specified - Process of using an eHealth tool | DHIs (e-health); General population | DHIs; health inequalities, ethnicity included in model as a demographic factor | Sets out steps of process of e-health tool use and where barriers could be addressed |
| Leach and Segal (2011) | Patient attributes warranting consideration in clinical practice guidelines, health workforce planning and policy | New framework | Workforce Evidence-Based model for diabetes mellitus | Chronic disease management (diabetes); Australia | Diabetes (cardiometabolic disease) | Support clinicians, researchers, etc. to recognise and effectively manage complex needs of individual patients with chronic disease |
| Li (2021) | Healthcare at Your Fingertips: The Acceptance and Adoption of Mobile Medical Treatment Services among Chinese Users | Adaptation of existing framework(s) | No name provided | 27 regions of China, primarily city-living | DHIs | Include additional factors - trust, privacy, personalisation, interactivity |
| Lowe et al (2015) | A Change for the Better? Digital Health Technologies and Changing Food Consumption Behaviors | New framework | Consumer adoption of DHTs | General | DHIs | Adoption of DHIs by individuals for food consumption behaviours |
| Yang Meier et al (2020) | Wearable Technology Acceptance in Health Care Based on National Culture Differences: Cross-Country Analysis Between Chinese and Swiss Consumers | Adaptation of existing framework(s) | Adapted WTAH model' (unnamed) | China and (German) Switzerland | DHIs (wearables) | To consider cultural(national) factors affecting acceptability of wearable technology |
| O'Connor et al (2016) | Understanding factors affecting patient and public engagement and recruitment to digital health interventions: a systematic review of qualitative studies | New framework | DIgital Health EnGagement MOdel (DIEGO) | DHIs(Qualitative studies of engagement and recruitment); General (no specific cohort) | DHIs | Model of patient/public processes and factors affecting decision-making and operationalising DHIs |
| Ondiege et al (2017) | Investigating User Identification in Remote Patient Monitoring Devices. | Adaptation of existing framework(s) | Senior Patients Technology Acceptance Model | Older adults living in homes where a single device needs to be shared with multiple users | DHIs | to describe acceptance and adoption of technology by elderly patients |
| Opoku et al (2017) | A realist review of mobile phone-based health interventions for non-communicable disease management in sub-Saharan Africa | Adaptation of existing framework(s) | Framework for understanding the contribution of mHealth interventions to improved access to care for patients with NCDs in sub-Saharan Africa | General | DHIs | A look at how mhealth interventions can be used to treat non-communicable diseases in sub-Saharan Africa |
| Perski et al (2017) | Conceptualising engagement with digital behaviour change interventions: a systematic review using principles from critical interpretive synthesis | New framework | Conceptual framework of direct and indirect influences on engagement with DBCIs | No geographical limitation; studies about engagement with DCBIs | DHIs (digital behaviour change interventions) | develop a conceptual framework specifying potential direct and indirect influences on engagement with digital behaviour change interventions (DBCIs) and relationships between engagement and intervention effectiveness |
| Putri et al (2020) | The antecedents and consequences of e-health literacy in the pharmaceutical industry: An agenda for future research | Adaptation of existing framework(s) | No name provided | South east Asian populations (although framework itself quick high level) | DHIs | to develop and validate a proposed conceptual framework for digital health literacy |
| Puuronen et al (2010) | A holistic framework for understanding acceptance of Remote Patient Management (RPM) systems by non-professional users | New framework | Framework for Studying user acceptance of RPM | Chronic disease patients | DHIs | User acceptance of remote patient management (RPM) systems |
| Salisbury et al (2015) | TElehealth in CHronic disease: mixed-methods study to develop the TECH conceptual model for intervention design and evaluation | New framework | TECH model (TElehealth in CHronic Disease) | Chronic disease management; telehealth | DHIs; CVD (chronic health) | Effective use of telehealth in management of chronic health conditions |
| Sari et al (2018) | A Proposed Conceptual Framework for Mobile Health Technology Adoption Among Employees at Workplaces in Malaysia | Adaptation of existing framework(s) | No name provided | Malaysian workers | DHIs | Mhealth user acceptance |
| Schillinger et al (2020) | The Intersections Between Social Determinants of Health, Health Literacy, and Health Disparities | New framework | Framework for the pathways that connect social determinants of health, health literacy and health disparities | USA general population | Health inequalities | Social determinants of health, health literacy and health disparities |
| Solar and Irwin (2010) | A Conceptual Framework for Action on the Social Determinants of Health | New framework | CSDH (Commission on Social Determinants of Health) conceptual framework | General | Health inequalities (social determinants of health) | Seeks to make clear the "distinction between the social causes of health and the social factors determining the distribution of these causes" - draws out structural and intermediary determinants |
| Su et al (2020) | Factors Affecting Patients' Acceptance of and Satisfaction with Cloud-Based Telehealth for Chronic Disease Management: A Case Study in the Workplace | Adaptation of existing framework(s) | No name provided | Taiwanese patients who each had participated in the telehealth program for at least 3 months | DHIs | to use for evaluating telehealth services in the workplace |
| Szinay et al (2021) | Perceptions of factors influencing engagement with health and wellbeing apps: a qualitative study using the COM-B model and Theoretical Domains Framework | Adaptation of existing framework(s) | Adapted TDF/COM-B (unnamed) | Adults (UK); Health and wellbeing apps | DHIs (health and wellbeing, general) | Consider factors influencing engagement with health and wellbeing apps |
| Tavares et al (2016) | Electronic Health record portal adoption by health care consumers | Adaptation of existing framework(s) | New research model | Patients | DHIs | Understand the factors which lead to the adoption of EHR portals |
| Venkatesh et al (2003) | User Acceptance of Information Technology: Toward a Unified View | Adaptation of existing framework(s) | Research Model | General | DHIs | User acceptance of DHIs |
| Wildenbos et al (2018) | Aging barriers influencing mobile health usability for older adults: A literature based framework (MOLD-US) | Adaptation of existing framework(s) | MOLD-US | Older adults | DHIs | a framework of these barriers and complexities associated with chronic diseases, and their potential impact on specific usability aspects of mHealth |
| Wilkinson et al (2016) | Meeting the Challenge of Diabetes in Ageing and Diverse Populations: A Review of the Literature from the UK | New framework | unnamed - theoretical framework from mapping of literature | Ethnic minorities; older people; diabetes; UK | Diabetes care; health inequalities | To provide framework to identify key areas for reducing inequalities in diabetes care for older people from ethnic minority groups. |
| Zhang et al (2021) | Theory integration for lifestyle behavior change in the digital age: An adaptive decision-making framework | New framework | Adaptive decision-making framework | Theory integration for lifestyle behaviour change DHIs (no specific population) | DHIs | to incorporate both traditional and more recent theoretical ideas about behaviour change in a single framework and reinterpret these ideas in light of a fine-grained temporal perspective |
| Zhao et al (2018) | What factors influence the mobile health service adoption? A meta-analysis and the moderating role of age | New framework | Mobile health services adoption framework | older people | DHIs | to develop a comprehensive framework regarding the adoption of individual mobile health services |
| Zhou et al (2019) | Factors influencing behavior intentions to telehealth by Chinese elderly: An extended TAM model | Adaptation of existing framework(s) | No name provided | Older people, China | DHIs | Including perception of service quality and behavioural intentions |

Abbreviations found in table:

CCM Chronic Care Model

CFIR Consolidated Framework for Implementation Research

CMD Cardiometabolic disease

CSDH Commission on Social Determinants of Health

DBCI Digital Behaviour Change Interventions

DHEF Digital Health Equity Framework

DIEGO DIgital Health EnGagement Model

DHI Digital Health Intervention

EHR Electronic Health Record

FITT Fit between Individuals, Task, and Technology (FITT) framework

HCP Health Care Professional

HITAM Health Information Technology Acceptance Model

HIV Human immunodeficiency Virus

ICTAM Information and Communication Technology Acceptance Model

IoT Internet of Things

KSA Kingdom of Saudi Arabia

NASSS Nonadoption, Abandonment, Scale-up, Spread, and Sustainability (NASSS)

NCD Non-Communicable Disease

RPM Remote Patient Management

TAM Technology Acceptance Model

TDF Theoretical Domains Framework

UK United Kingdom

USA United States of America

UTAUT Unified Theory of Acceptance and Use of Technology

WTAH Wearable Technology Acceptance in Health
